# Hip Pain and Fever During a Nadir Period: Who Came First?

**DOI:** 10.7759/cureus.48697

**Published:** 2023-11-12

**Authors:** Miguel Reis Costa, Ângela Ferreira, Catarina Carvoeiro, Irene Miranda, Diana Guerra

**Affiliations:** 1 Internal Medicine, ULSAM - Hospital de Santa Luzia, Viana do Castelo, PRT

**Keywords:** fever, abscess, nadir, chemotherapy, pyomyositis

## Abstract

Pyomyositis is a rare bacterial infection of the skeletal muscle that often presents with insidious symptoms, thus making the diagnosis challenging. It is categorized into primary and secondary (usually traumatic) variants, mainly occurring in tropical regions and occasionally in temperate climates, with predisposing factors including immunosuppression. Staphylococcus aureus is the most common pathogen. A 39-year-old woman with a history of breast cancer underwent a mastectomy followed by chemotherapy. After her second chemotherapy cycle, she developed fever, odynophagia, vomiting, and right hip pain (considered to be related to muscle tension due to functional exercises). Fever and hip pain progressively worsened and the other symptoms resolved. On the 12th day after chemotherapy, she received intramuscular diclofenac injections due to severe hip pain. Physical examination revealed tenderness in her right hip and signs of inflammation on her thigh and buttock. Laboratory tests showed elevation of inflammatory markers and mild kidney and liver dysfunction. A CT scan revealed an intramuscular collection in her right gluteal region (~45 x 70 mm), with adjacent fat densification. Attempts to drain the collection initially failed, but a later ultrasound-guided procedure was successful and pus was collected for bacterial culture, which identified methicillin-susceptible Staphylococcus aureus (MSSA). Antibiotic treatment was adjusted to target SA with flucloxacillin and the patient's condition improved. Subsequent imaging showed a resolving collection (<10 mm). The patient continued antibiotic treatment for six weeks, maintaining clinical improvement, normal inflammatory parameters, and apyrexia. Adjuvant chemotherapy was discontinued due to the risk of infection recurrence associated with the multiloculated collection caused by SA. The patient remained asymptomatic four months after hospitalization. An MRI then showed only a residual T2 hyperintensity in the deeper region of the right buttock, with no visible collections. The nadir period refers to the time after each chemotherapy cycle when the risk of neutropenia and subsequent infection is the highest, typically occurring between 7 and 14 days after each cycle. In this case, the intramuscular injection occurred 12 days after the second cycle of chemotherapy. It is most likely that this served as the entry point for the pyomyositis agent (MSSA) during a period of transient neutropenia.

## Introduction

Pyomyositis is a bacterial infection of the skeletal muscle that can form abscesses and/or spread locally. It often has an insidious course, and diagnosis can be challenging due to its rarity, vague initial symptoms, and low specificity of diagnostic tests. Pyomyositis is divided into primary (tropical), which occurs via hematogenous spread with presumed or confirmed bacteremia, and secondary, which results from penetrating trauma or contiguous spread from adjacent infected tissues. It is mainly reported in tropical regions of Asia, Africa, Oceania, and the Caribbean islands [1,2,3].

In temperate countries, it is a rare condition, affecting approximately 0.5 cases per 100,000 people per year, primarily as a secondary condition often precipitated by trauma or vigorous physical activity. There are intrinsic predisposing factors, including immunosuppression, chronic illness, intravenous drug use, and recent infections (bacteremia, pneumonia, tuberculosis, chickenpox, etc.) [4]. About 75% of reported cases occur in immunocompromised individuals. Staphylococcus aureus (SA) is the most frequently associated pathogen, with studies suggesting it accounts for more than 90% of cases in tropical areas and over 75% in temperate regions [1,2,4]. Included are strains of methicillin-resistant Staphylococcus aureus (MRSA) and certain strains of methicillin-sensitive S. aureus (MSSA) in the community (particularly ones that have acquired the Panton-Valentine leukocidin virulence factor) - these demonstrate a tendency for aggressive and disseminated infections [5].

## Case presentation

A 39-year-old Caucasian woman, with a history of multifocal invasive ductal carcinoma grade 2 of the right breast (T1N0M0 R0), with hormonal receptor positive (with both estrogen receptor-positive 90-100% and progesterone receptor-positive 90-100%), Ki67 20% and HER2 negative, underwent a subcutaneous right mastectomy, followed by adjuvant chemotherapy with docetaxel + cyclophosphamide (including dexamethasone before each cycle). On the 10th day after the second (and final) cycle of chemotherapy, she developed odynophagia and fever (maximum 38.5°C) with chills. On the 11th day, she experienced nausea and vomiting; on the 12th day, she developed hip pain (right>left). She mentioned engaging in repeated functional physical activity, including a few squats, involving the joint in the preceding days. On this day, due to intense pain upon mobilization and touch in the trochanteric region, she received two intramuscular doses of diclofenac (50 mg each dose) in the upper lateral quadrant of the gluteus. Meanwhile, her odynophagia and diarrhea resolved, but she continued to have a fever, and the hip pain worsened: primarily located in the right trochanteric and buttock area, radiating halfway down the lateral thigh. She reported increased pain without stiffness in the morning upon waking up. She denied urinary symptoms, headache, contact with sick individuals, exposure to non-dewormed animals, contaminated food, and trips to tropical regions. On physical examination in the Emergency Department, the patient appeared neurologically intact (without meningeal signs) and normotensive, but with sinus tachycardia (125 bpm). Her tympanic temperature was 37.3°C (under the influence of 1 g paracetamol). Examination revealed tenderness upon palpation of the right greater trochanter with mobilization of the right hip joint and dorsiflexion of the foot, with no further inflammatory signs. No abnormalities were found in the contralateral limb, and there were no other physical examination findings, including normal cardiopulmonary auscultation. Laboratory tests showed mild anemia (present since the start of chemotherapy), acute kidney injury (creatinine 1.77 mg/dL, baseline 0.8 mg/dL), low-grade cholestasis (gamma-glutamyl transferase 101 UI/L; alkaline phosphatase 121 U/L; AST/ALT: 59/49 UI/L and total bilirubin 1.51 mg/dL with direct bilirubin 0.93 mg/dL), mild liver dysfunction with coagulopathy - international normalized ratio (INR) of 1.5, and significantly elevated C-reactive protein (CRP) and procalcitonin (PCT) - 44 mg/dL and 7.6 ng/dL, respectively. Urinalysis showed six leukocytes per field. Blood gas analysis showed no significant abnormalities. Respiratory panel for influenza A, respiratory syncytial virus (RSV), and SARS-CoV-2 and septic workup (three blood cultures and one urine culture) were negative. A non-contrast thoracoabdominopelvic CT scan was performed and did not show suggestive infectious foci. Ultrasound (US) with Doppler of the right lower limb revealed no signs of deep vein thrombosis and no intra-articular effusion, excluding inflammatory trochanteric bursitis. The patient had no clinical or imaging findings compatible with septic arthritis. She was admitted to the ward and started receiving empiric intravenous ceftriaxone 2 g and fluid therapy. Due to suspicion of femoral head osteonecrosis, a right thigh MRI was requested for inpatient evaluation.

On the third day of treatment, the patient continued to have a fever, maintaining CRP levels, showing the emergence of severe leukocytosis >20.000 cel/mm^3^ with neutrophilia. Table 1 summarizes the variation of inflammatory and muscle markers during hospitalization.

**Table 1:** Inflammatory and muscle markers during the course of the disease. Although CRP and procalcitonin levels have been elevated from the beginning, leukocytosis is only observed from the third day of hospitalization. This is due to the delayed immune response following the nadir period after chemotherapy. On the other hand, myoglobin is slightly elevated at the initial stage, and Ck was normal; in fact, in pyomyositis, the infection is usually contained within the muscle, and consequently the muscle damage may not be as extensive as in conditions such as rhabdomyolysis. Therefore, the release of muscle proteins into the bloodstream may not be as significant, leading to relatively normal myoglobin/Ck levels in the blood.

	Admission	Day 3	At discharge
Leukocytes (<10.000/uL)	8.410/uL	23.970/uL	5.320/uL
Neutrophils (<80%)	78%	87%	63%
CRP (<1 mg/dL)	44.6 mg/dL	30.36 mg/dL	0.74 mg/dL
Procalcitonin (<0.05 ng/dL)	7.61 ng/dL	1.99 ng/dL	0.04 ng/dL
Myoglobin (<90 ng/mL)	92 ng/dL	119 ng/dL	22 ng/dL
Creatine kinase (Ck) (<294 U/L)	286 U/L	330 U/L	40 U/L

**Figure 1:**
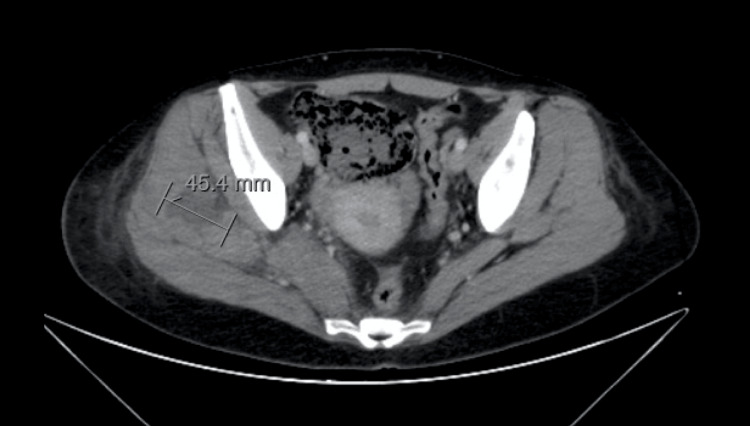
Pelvic CT scan performed on the third day. The contrasted CT scan revealed a multiloculated intramuscular collection in the right gluteal region, approximately 45 x 70 mm in size.

Vancomycin was added to the treatment (targeting 15-20 mcg/dL). Fever spikes became less frequent. The patient continued to experience worsening right hip pain, with signs of inflammation appearing on the lateral thigh and buttock. Therefore, a repeat abdominal-pelvic CT scan with extension to the lower limb was performed (by this time, renal function had normalized). As we can see in Figure 1, it revealed an extensive multiloculated intramuscular collection in the right gluteal region, approximately 45 x 70 mm in size, with adjacent fat densification.

**Figure 2:**
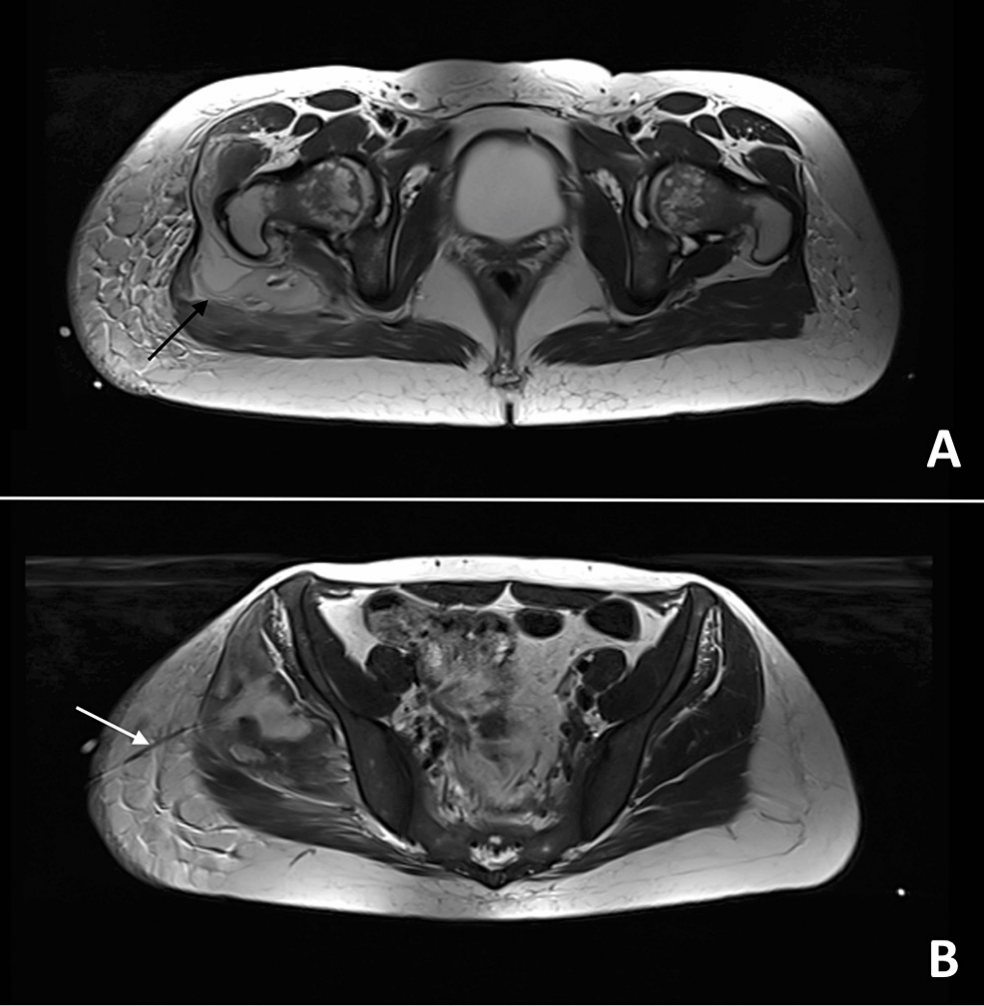
Pelvic and upper right leg MRI scan (T2): transverse plane (image A) and coronal plane (image B). In image A, we can confirm the bigger intramuscular collection (black arrow) in the right gluteal with higher resolution (compared to a CT scan) of adjacent fat densification. In image B, it was also possible to check the correct placement of the drain (white arrow). In this same MRI (in more distal sections), it was also possible to discover a new isolated thick-walled liquid collection, measuring 13 mm in the muscle belly of the rectus femoris (proximal third of the thigh), suggestive of an abscess.

An attempt was made to drain the fluid for microbiological analysis, but it was unsuccessful (the density was similar to muscle at this stage - thick fluid). On the fifth day of hospitalization, an US reevaluation of the collection was performed, and due to the lower fluid density at this point, drainage of the purulent collection became feasible. Pus was collected for bacteriological culture. By this time, blood and urine cultures were negative. On the sixth day, a follow-up MRI was finally performed, confirming the correct placement of the drain and revealing a new isolated thick-walled liquid collection, measuring 13 mm in the muscle belly of the rectus femoris (proximal third of the thigh), suggestive of an abscess. Figure 2 shows a tranverse plane (image A) and a coronal plane (image B) of this MRI scan.

On the ninth day, SA was isolated from the pus, prompting the discontinuation of ceftriaxone (vancomycin was continued), and on the 10th day, methicillin sensitivity was confirmed, leading to a switch in antibiotic therapy to flucloxacillin 2 g every six hours. A transthoracic echocardiogram was performed, showing no signs of infective endocarditis. Inflammatory parameters continued to decrease, and the patient remained apyretic after the drain placement. On the 14th day, a US reevaluation showed favorable evolution, with the largest locule drained, and scattered smaller locules without indication for drain replacement. Therefore, the drain was removed (draining a total of 325 mL of pus). The patient was discharged with flucloxacillin 2 g every six hours and scheduled for an internal medicine consultation after two weeks. The final diagnosis was considered a pyomyositis of the right gluteus by MSSA, in an immunosuppressed patient.

**Figure 3:**
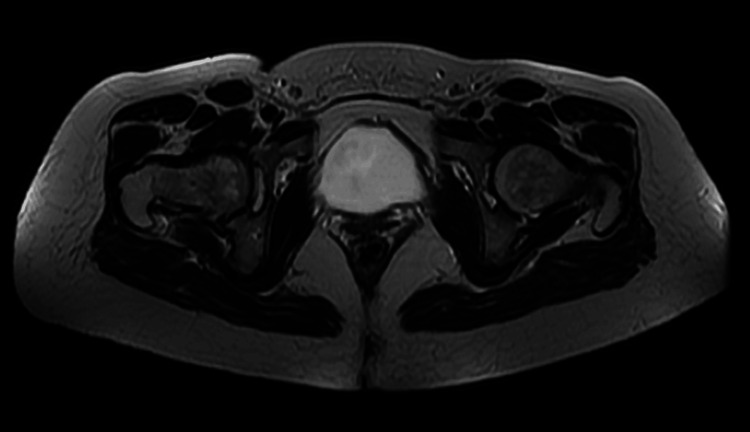
Pelvic and upper right leg low-resolution MRI scan (T2) performed four months after hospitalization. At this point, it was only visible a residual T2 hyperintensity in the deeper region of the right buttock, with no visible collections.

At the follow-up consultation, the patient was afebrile, reported improvement in pain and functional capacity of the limb, and had normalized inflammatory parameters (CRP 0.4 mg/dL). At this point, a repeat CT scan of the lower limb with contrast showed a "mild densification in the ischiofemoral space, currently observing a thin liquid collection with 8 mm thickness, without drainage criteria." The decision was made to continue antibiotic therapy for an additional two weeks; however, due to marked gastrointestinal complaints, flucloxacillin was discontinued, and cotrimoxazole 960 mg twice a day was initiated (by this time, effective antibiotic therapy had been administered for four weeks). The patient completed six weeks of antibiotic therapy without complications, maintaining gradual clinical improvement, apyrexia, and sustained low inflammatory parameters. Considering the cost-benefit ratio, a multidisciplinary team decision was made to discontinue adjuvant chemotherapy. The patient continued to be monitored by internal medicine, and four months after hospitalization, she was asymptomatic with no functional deficits. At this time, a low-resolution MRI scan showed only residual T2 hyperintensity in the deeper region of the right buttock, with no visible collections. Figure 3 reveals a T2 frame of this last MRI scan where a complete resolution of the right gluteal intramuscular collection is visible in previous Figure 2 (image A).

## Discussion

The nadir period refers to the time after each chemotherapy cycle when the risk of neutropenia and subsequent infection is the highest, typically occurring between seven and 14 days after each cycle [6]. In this case, although there was no laboratory confirmation of neutropenia during this period, the authors know that the intramuscular injection that occurred 12 days after the second cycle of chemotherapy poses a significant risk mainly due to preexisting surface colonization of bacteria. It is most likely that this served as the entry point for the pyomyositis agent (MSSA) during a period of transient immunosuppression. Remember that, on day three of antibiotic therapy, there was a severe increase in leukocytosis with CRP regression, which is explained by the immunological state of the patient at the end of the nadir period with a “late” response to infection. This thought is consistent with the idea that the first bilateral pain of the patient was caused by stress/traumatic and inflammatory trochanteritis and the initial low-grade fever and that transitory flu symptoms were probably due to a simple upper respiratory viral infection in the early stage of the nadir period.

The natural history of pyomyositis includes three stages: invasive, purulent, and late stage [3]. At the invasive stage, the patient experiences insidious onset of dull, crampy, localized muscle pain, followed one week later by edema, worsening pain, and low-grade fever. A mild leukocytosis may be present. The examination reveals few signs of inflammation with minimal swelling or fluctuance. Patients presenting 10-21 days from the onset of symptoms are in the purulent stage. They often display marked edema and tenderness of the muscle, associated with fever and other signs of inflammation; marked leukocytosis is usually present. It is often at this stage that the patients are diagnosed (>90%). The late stage is characterized by systemic toxicity with high fever. In the reported case, the authors consider that the patient was admitted in transition between the invasive and purulent stages, based on the evolution of the clinic (pain and fever), leukocytosis, and radiographic imaging.

Laboratory findings are nonspecific: elevated ESR and CPR and neutrophilic leukocytosis with a left shift in all cases. White blood cell count is elevated in only 19% of immunocompromised patients [7]. Counterintuitively, muscle enzymes, such as creatine kinase, myoglobin, and aldolase levels, are normal or only minimally elevated. The diagnosis of pyomyositis relies on imaging. US is a relatively accessible, non-invasive, radiation-sparing modality that can diagnose an abscess. When ultrasound is inconclusive, CT or MRI is indicated. MRI with gadolinium imaging is highly sensitive to an abscess, muscle inflammation, and infection of adjacent structures because it can image large areas of the body and detect subclinical involvement [8].

The basis of treatment of pyomyositis is antibiotic therapy combined with surgical or image-guide percutaneous drainage for patients who develop an abscess. Empirical antimicrobial therapy will depend upon local epidemiologic and susceptibility patterns. Broad-spectrum antibiotics covering SA and Streptococcus are appropriate. In our institution, high-dose IV flucloxacillin is the empiric antibiotic of choice. In patients with known or suspected MRSA, vancomycin or clindamycin treatment must be considered. In immunocompromised patients, Gram-negative organisms should be covered [1,2,4]. This case reported an initial administration of empiric ceftriaxone although no infection foci were identified.

Optimizing treatment depends on identifying the causative agent. In temperate regions, blood cultures are positive in only 30% of the cases, and organisms are usually identified from abscess aspirates [1], as occurred in the reported case. This allowed us to adjust the treatment to flucloxacilin. Most patients with pyomyositis require a total of four weeks of antibiotic therapy [2]. In the reported case, the decision to prolong antibiotic therapy for a maximum of six weeks was based on the presence of a small collection that was not amenable to drainage. Because of the experienced gastrointestinal side effects due to flucloxacillin, the antibiotic was switched to cotrimoxazole in the two final weeks. It should not be used in the early stages of this infection because infections with a lot of inflammatory tissue such as abscesses produce significant amounts of thymidine, which may inactivate trimethoprim-sulfamethoxazole [9].

Since MRI often overestimates areas of inflammation, a decision was made to perform a repeat MRI only a few months after the end of this antibiotic therapy period. During the initial follow-up, CT was used for monitoring. This case is an example that the decision concerning the duration of antibiotic treatment in pyomyositis should consider the clinical status and inflammation markers and not only the imaging evaluation. For example, MRI overestimates inflammatory signs even when the infection is resolved, and these signs frequently are only related to healing tissue [10].

The multidisciplinary approach, in this case, was essential for discontinuing chemotherapy: on the one hand, internal medicine raised concerns that the multiloculated collection caused by an aggressive microorganism such as SA would carry a significant risk of infection recurrence [11]; on the other hand, according to medical oncology, as a patient who had undergone mastectomy with clear margins and completed two cycles of adjuvant chemotherapy, her benefit from further treatment would be minimal [12].

## Conclusions

Due to its rarity in temperate countries, slow evolution, and non-specific diagnostic auxiliary tests in the initial phase, pyomyositis is a challenging diagnosis. It should always be considered when there is fever and pain restricted to a muscle group, especially when there is immunosuppression and a history of trauma. This case highlights the relationship between focus control and prognosis, as well as the importance of the duration of antibiotic therapy, which should be individualized and based on clinical response.
